# Hubs of knowledge: using the functional link structure in Biozon to mine for biologically significant entities

**DOI:** 10.1186/1471-2105-7-71

**Published:** 2006-02-15

**Authors:** Paul Shafer, Timothy Isganitis, Golan Yona

**Affiliations:** 1 Department of Computer Science, Cornell University, Ithaca, NY, USA

## Abstract

**Background:**

Existing biological databases support a variety of queries such as keyword or definition search. However, they do not provide any measure of relevance for the instances reported, and result sets are usually sorted arbitrarily.

**Results:**

We describe a system that builds upon the complex infrastructure of the Biozon database and applies methods similar to those of Google to rank documents that match queries. We explore different prominence models and study the spectral properties of the corresponding data graphs. We evaluate the information content of principal and non-principal eigenspaces, and test various scoring functions which combine contributions from multiple eigenspaces. We also test the effect of similarity data and other variations which are unique to the biological knowledge domain on the quality of the results. Query result sets are assessed using a probabilistic approach that measures the significance of coherence between directly connected nodes in the data graph. This model allows us, for the first time, to compare different prominence models quantitatively and effectively and to observe unique trends.

**Conclusion:**

Our tests show that the ranked query results outperform unsorted results with respect to our significance measure and the top ranked entities are typically linked to many other biological entities. Our study resulted in a working ranking system of biological entities that was integrated into Biozon at .

## Background

There is vast amount of heterogeneous biological data today that is warehoused in multiple databases. Among these are databases of protein sequences [1,2], protein structures [3], DNA sequences [4], protein-protein interactions [5,6], cellular pathways [7,8], and many others. These databases are typically highly focused and are usually limited to one data type. However, biological entities are strongly related and mutually dependent on each other, and to properly analyze the function of an entity one needs to know its extended biological context and its relation to other entities. For example, to define most accurately the functional role of a specific gene it is necessary to consider also the interactions it is involved in and the set of biochemical pathways it participates in. This mutual dependency is especially important when, for example, querying this wealth of data for disease related genes or for interactions that mediate signal transduction in a specific biological system. The integration of information from multiple resources can either corroborate a certain aspect of a biological entity, validate a hypothesis, or sometimes give initial clues to the function of a completely uncharacterized object.

Existing methods for querying biological data available on the web are mostly limited to the one data type warehoused in the database being queried. There are a few servers that allow one to query multiple databases at once, such as the NCBI entrez server [9] and the EMBL server [10]. These servers also maintain links among different entities stored within. Other servers such as Moby [11] and Biomediator [12] provide interface to query multiple resources. However, all these servers do not integrate the results or analyze the relations between the objects at query time. Moreover, the query results are ordered arbitrarily or by features irrelevant to the query (e.g. in alphabetical order). This is clearly not ideal as one might need to scan through hundreds or thousands of matches before encountering the instance that is the most relevant, the most studied, or the most interconnected. Furthermore, there are many instances in biological databases that are partially annotated or completely uncharacterized. Even if biologically relevant to the search term, these objects will be overlooked by traditional search methods. However, the relations between these objects and other, better annotated objects may help identify their functions. This may in turn imply that these objects are indeed relevant to the search term.

There has been a substantial amount of work directed towards developing methods and **prominence models **for effectively querying and ranking documents on the World Wide Web. The underlying idea behind many prominence models is that the link structure of the Internet can be used to identify the web pages most relevant to the user's query. A well-known example is Google's PageRank [13]. Another model, proposed in [14], identifies "hubs" and "authorities," where authorities are web pages that are linked to by prominent hubs and hubs are pages that link to prominent authorities. Both models assign prominence values to documents using the eigenvectors of the data graph's adjacency matrix (or a close variant). Many studies have tested variants of these models; for a review the reader is referred to [15,16]. A recent study has generalized link-based ranking techniques developed for the Web to arbitrary relational databases [17] by analyzing relations induced by a set of queries. This work ranks data instances through a spectral analysis of what they call a database graph, defined in terms of a database and a (finite) set of queries (query language). In this graph a vertex corresponds to a tuple of data and two tuples are related by an edge if there is a query in the query language that outputs one when using the other to specify the query's parameters. However, as the authors indicate, the rankings produced by this model are sensitive to the choice of query language.

Today's most advanced systems belong to companies such as Google, Yahoo and Microsoft. Unfortunately, these companies do not disclose the algorithmic details of their search and ranking engines which augment the link analysis with information retrieval (IR) techniques. But despite the significant progress in ranking web documents, no equivalent systems were developed in the biological knowledge domain. The only exception is PubMed [18] that uses IR techniques to identify documents that are related to a given document, based on their similarity of word frequency.

This paper addresses the problem of how to query *heterogeneous biological data *effectively. We sort query results based on their biological significance by exploiting the relationships between biological entities. Unlike a web query, when querying heterogeneous biological data, a user might be interested in all entities (of one or more data types) that match the query and in the relationships among these entities. We refer to these sets of interconnected instances that share a common theme as **hubs of knowledge. **Detecting these hubs is the main focus of this paper.

We propose a model which resembles methods that are used to search and rank web documents. Our method builds on the extensive schema of the Biozon database, the heterogeneous data stored in this database, and its link structure that connects different biological entities. This link structure is used both to propagate information between documents and to rank matches based on their broader biological context. We explore several different approaches and prominence models, study the properties of these systems, and develop means to evaluate their effectiveness and compare their performance using an objective probabilistic model. We study the effect of non-principal eigenspaces on the ranking and test several different functions for combining their contributions. We also test variants that consider the special properties of the biological data network. Specifically, in the Biozon setting, each link carries a specific meaning with it, and certain relations are deemed more significant than others. Therefore, the models that are used to rank web documents have to be modified to query biological data effectively. We test variants that account for similarity relationships between entities and for their statistical significance, and we test other variants that consider also the semantic significance of other relations.

This paper is organized as follows. We start with a brief description of the Biozon database, the prominence models we tested and the different search strategies. Next we present several example queries and their results followed by qualitative conclusions ('Results'). We proceed to a rigorous performance evaluation of the proposed prominence models and of the use of non-principal eigenspaces ('Discussion'). A few variants are described next and are followed by conclusions.

## Results

### The Biozon database and data graph

Biozon is a system that consolidates multiple biological databases consisting of a variety of heterogeneous data types (such as DNA sequences, proteins, interactions and cellular pathways) into a single extensive schema that is logically represented as a large **data graph **Σ. Each node represents some datum, and an edge between two nodes represents a relationship between them. Formally, Σ = (**D**, **R**) where **D **= {*d*_1_*...d*_*n*_} is the set of all nodes (documents) in the graph and **R **= {*r*_1_*...r*_*m*_} ⊂ **D **× **D **is the set of all edges (relations) in the graph. Much of the data in Biozon is gleaned from publicly available databases such as SwissProt, PDB, GenBank, BIND, KEGG, and more. In addition, Biozon stores novel computed data, such as similarity relationships and functional predictions. The data is warehoused locally so the fundamental biological objects represented are *non-redundant *even though data within and between their originating sources overlap. The Biozon resource is available online at .

In this study we consider the following subset of the Biozon data types: nucleic acid sequences, protein sequences, protein structures, enzyme families, interactions, and pathways. We also consider all relations among these types, including the relations 'member of', 'manifests as', 'encodes', 'similar', and 'contains' (for more information on the Biozon data and relation types, see subsection 'The Biozon database and data graph' of the 'Methods' section). This subgraph, at the instance level, is the subject of our analysis of prominence models.

### Prominence models

All the prominence models we consider are based on the idea that a node is prominent if it is connected to other prominent nodes. Given a query, our analysis starts by defining the graph (or subgraph) of relevant documents and its **adjacency matrix A. **From the adjacency matrix we derive a **connectivity matrix B, **using some function. The spectral properties of the connectivity matrix are then analyzed by computing its eigenvectors. Each eigenvector is considered to be a possible assignment of prominence values to documents, where node *u *is assigned a prominence value equal to the *u*^*th *^component of the eigenvector. The highest scoring nodes in the principal vector(s) are returned as potential significant documents that match the query.

The spectral methods we consider in this paper are based on those used to rank documents on the World Wide Web. There are, however, several important differences between the structure of the Internet and that of the Biozon database. First, the hyperlink structure of the Internet defines a clearly directed graph using a single type of relation (the "links to" relation). In contrast, we generally view the Biozon data graph as undirected, unless we impart a specific, biologically motivated direction associated with the various types of relations (see subsection 'Forced directed graphs' in the 'Methods' section). Second, the nodes of the Internet are generally viewed as a homogeneous set, while Biozon's objects represent heterogeneous biological entities.

We test four main methods for assigning prominence values to nodes: Eigenvector Centrality, Hubs & Authorities, PageRank and Katz's Status. The methods differ in the way they characterize the connectivity among the elements of the subgraph. First we make the distinction between *sparse *and *dense *models. Eigenvector Centrality, Hubs & Authorities and Katz's Status are **sparse models **in the sense that they analyze connectivity matrices that are derived directly from the adjacency matrices of sparse graphs and hence are of the same order of sparsity. PageRank on the other hand augments the adjacency matrix with a complete matrix of prior probabilities, and hence practically analyzes a new, completely connected graph. Among sparse models, the differences are subtle, but for undirected graphs they are essentially reduced to the paths that are considered by the model. For example, Eigenvector Centrality takes the connectivity matrix to be simply the adjacency matrix, while Hubs & Authorities defines the connectivity matrix based only on paths of length two between objects. Katz's Status combines information from all paths of length one, two and three. The four models are described in more detail in the 'Methods' section.

In the 'Discussion' section we explore several variations of these methods that test the effect of non-principal eigenspaces on the ranking, different weighting functions, and inclusion of similarity data in the adjacency matrix.

### Computing prominence vectors

In the prominence models we considered, prominence values are associated with the eigenvectors of the corresponding connectivity matrices. As such, any method for finding eigenvectors will suffice. A special case is multidimensional eigenspaces. We take the prominence of an object *i *in an eigenspace with cardinality *k > *1 to be the projection of that object on the eigenspace:

e^(i)=∑j=1kej(i)2
 MathType@MTEF@5@5@+=feaafiart1ev1aaatCvAUfKttLearuWrP9MDH5MBPbIqV92AaeXatLxBI9gBaebbnrfifHhDYfgasaacH8akY=wiFfYdH8Gipec8Eeeu0xXdbba9frFj0=OqFfea0dXdd9vqai=hGuQ8kuc9pgc9s8qqaq=dirpe0xb9q8qiLsFr0=vr0=vr0dc8meaabaqaciaacaGaaeqabaqabeGadaaakeaaieqacuWFLbqzgaqcaiabcIcaOiabdMgaPjabcMcaPiabg2da9maakaaabaWaaabCaeaacqWFLbqzdaWgaaWcbaGaemOAaOgabeaakiabcIcaOiabdMgaPjabcMcaPmaaCaaaleqabaGaeGOmaidaaaqaaiabdQgaQjabg2da9iabigdaXaqaaiabdUgaRbqdcqGHris5aaWcbeaaaaa@403A@

where {**e**_1_, **e**_2_, ..., **e**_*k*_} is an orthonormal basis of the eigenspace. For a more detailed discussion of computing prominence vectors see 'Methods'.

### Search strategies: global graph vs. focused subgraphs

**Figure 1:**
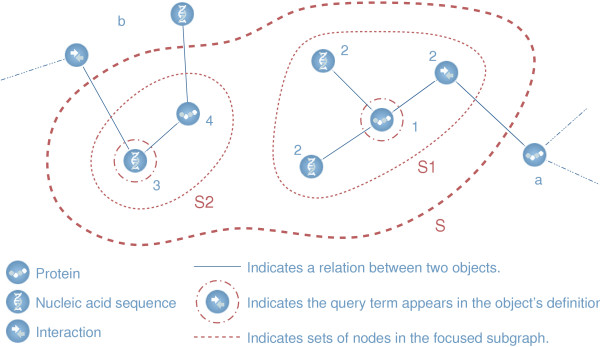
**Generating the focused subgraph**. Suppose we are searching protein definitions for ubiquitin in the above Biozon subgraph. Each of the two circled nodes corresponds to an entity in which "ubiquitin" appears in its definition field. The set **S **represents all nodes that will be included in the focused subgraph. These nodes are numbered according to the steps in which they are added to the focused subgraph (see 'Methods'). **S_1 _**is the set of all *proteins *that match the query term and their neighbors, while **S_2 _**is the set of all *non-proteins *that match the query term and their protein neighbors. Nodes are included in the subgraph if one of these two criteria is met: (a) A protein whose definition does not contain the search term is included only if it has a neighbor whose definition does contain the search term. (b) A non-protein whose definition does not contain the search term is included only if it has a *protein neighbor *whose definition does contain the search term.

Our ultimate goal is to provide a sensible ranking of results from queries to the Biozon database. We consider three general strategies for generating and then ordering these result sets: The **focused subgraph method **(referred to also as the 'local method') first creates a subgraph of Biozon consisting of nodes that satisfy the query and their immediate neighbors as described in Fig. 1. Prominence values are then assigned to the nodes of the subgraph using one of the above models, and nodes are ranked by descending prominence. The **global method, **on the other hand, first uses one of the models to assign prominence values to every node in the Biozon graph. Then, for a given search, nodes satisfying the query are extracted and ordered by descending prominence. The main advantage of the global method is its speed since the prominence values can be pre-computed. However, it is less sensitive than the local method which can report entities that do not match the query term but may still be relevant to the query (based on their relations with entities that do match the query). The **extended global **method is a variation of the global method which utilizes information in neighboring entities to bring forward uncharacterized entities, thus combining the advantages of both methods. For a detailed description of these procedures see section 'Search strategies' in 'Methods'.

### The spectral properties of the connectivity matrices

#### Examples

To demonstrate the effect of the different prominence models on the ranking of biological documents we ran several test queries. For each query we generated an undirected focused subgraph of the Biozon database as described in Fig. 1. From that subgraph we constructed four different connectivity matrices, one for each prominence model, and computed the eigenvectors corresponding to the twenty largest-magnitude eigenvalues for each matrix. After translating each multidimensional eigenspace to a single prominence vector (as described in section 'Computing prominence vectors'), we examined the 50 highest scoring documents in each of these prominence vectors.

In general, we observe that sparse models (Eigenvector Centrality, Hubs & Authorities, and hybrid Katz's Status) perform similarly in the sense that they commonly emphasize the same biological entities in each of their respective eigenspaces. If Eigenvector Centrality ranks some object in a particular eigenspace highly, then Hubs & Authorities and hybrid Katz's Status also rank that object highly in the corresponding or in a nearby eigenspace. (Corresponding Eigenvector Centrality eigenspaces and Hubs & Authorities eigenspaces emphasize the same biological entities. Hybrid Katz's Status eigenspaces may be off by one or so.) PageRank, on the other hand, tends to consolidate the most significant information from across the other methods' eigenspaces into its principal eigenspace.

For example, we searched for proteins with the definition ubiquitin. These proteins are involved in protein degradation and are attached to other proteins by an enzyme, thereby marking them for degradation. Sparse models rank the protein family ubiquitin-protein ligase, ubiquitin-activating enzyme as the most prominent entity in the *principal *eigenspace (This family is associated with Biozon docid 5977355. To view an entry with docid *x*, follow the URL http://www.biozon.org/Biozon/Profile/x). The family is followed by its members, such as Ubiquitin-like protein SUMO-l conjugating enzyme (docid: 365280), Ubiquitin-protein ligase RSP5 (docid: 1026628), and Ubiquitin-activating enzyme (docid: 1054300). The most prominent entity in the *second *eigenspace is the protein family ubiquitin thioiesterase (docid: 5975715). It is followed by its members Ubiquitin carboxyl terminal hydrolase 15 (docid: 1066565), Ubiquitin carboxyl terminal hydrolase 6 (docid: 911606), and Ubiquitin carboxyl terminal hydrolase 14 (docid: 1025595). We observe that the most prominent proteins in these two eigenspaces are generally encoded by several DNA sequences and are involved in several interactions. Although these DNA sequences and interactions are not among the most prominent elements of these eigenspaces, they do contribute to the high ranking of the proteins. The third eigenspace emphasizes the protein Skplp (docid: 446186) and its interactions. This protein is involved in ubiquitin-mediated proteolysis and is part of a larger complex that underlies many of the interactions associated with it. The fourth eigenspace emphasizes protein orf yol133w (docid: 259185) and its interactions. Note that the main definition of this protein does not contain the query term.

However, interaction data suggests ubiquitin-related activity. The fifth through seventh eigenspaces behave similarly to the third and fourth eigenspaces. Each focuses on a protein and its interactions. The fifth eigenspace emphasizes UV excision repair protein RAD23 (docid: 808559) and 26S protease regulatory subunit 7 homolog (docid: 882149). Interestingly, the sixth eigenspace emphasizes these same two proteins, however, while the fifth eigenspace favors the first, the sixth eigenspace favors the latter.

The most prominent entities in PageRank's *principal *eigenspace are the two protein families mentioned above and their member proteins, as well as the proteins emphasized in the non-principal eigenspaces of the other methods. In general, PageRank tends to favor entities with a high degree. For example, proteins Ubiquitin carboxyl terminal hydrolase 15 (docid: 1066565) and Ubiquitin-activating enzyme (docid: 1054300) are among the 50 most highly ranked entities by PageRank and are members of the two families mentioned above. Both of these proteins have many neighbors (including multiple protein-protein interactions). These proteins are thus highly ranked on their own merit, and not just because they are members of a highly ranked protein family.

We also observe this phenomenon when using the query term cancer. For cancer, the most prominent entities in the principal eigenspaces of the sparse models are the tumor suppressor protein P53 (docid: 802537) and its interactions. The second eigenspace emphasizes protein breast cancer type 1 susceptibility protein (docid: 1079763) and its interactions. The third eigenspace emphasizes DNA repair protein RAD51 (docid: 811200) and its interactions. The fourth eigenspace emphasizes protein CRK-associated substrate (docid: 1036799), which is a breast cancer anti-estrogen resistance protein, and its encoding DNA sequences and interactions. PageRank's principal eigenspace emphasizes all four of these proteins. Similarly, for the query term autoimmune, we observe the following. The principal eigenspace of the sparse models emphasizes protein Autoimmune regulator (docid: 947655), protein Autoimmune regulator (docid: 943600) and Ribonuclease P protein subunit RPP1 (docid: 640274) along with the nucleic acid sequences that encode these proteins. The second eigenspace emphasizes Tumor necrosis factor ligand (docid: 621283) Tumor necrosis factor precursor (docid: 533673) and the DNA sequences that encode either of them. PageRank's principal eigenspace emphasizes all of the above proteins (besides 533673) along with several DNA sequences which encode for these and similar proteins.

We compare these results with those generated by similar queries to the NCBI or SwissProt servers. For example, given the query ubiquitin, both servers only return records that contain the query term. The SwissProt set is ordered alphabetically based on the protein ID, while the NCBI server orders the records based on the date the record was created. Both orderings are quite arbitrary and uninformative. On the other hand, the examples described above demonstrate that the prominence models we tested are indeed effective in identifying interesting instances that are highly connected to other biological entities, thereby providing a broader biological context for functional analysis of these instances. Our observations of independent queries suggest that sparse models most readily identify *Hubs of Knowledge *as different eigenspaces tend to emphasize nearly disjoint sets of intrarelated biological entities. Consequently, no single eigenspace completely characterizes the set of most important entities in the focused subgraph. On the other hand, PageRank's principal eigenspace does appear to summarize this set by singling out the highest scoring entities in each of the other methods' top eigenspaces. Thus PageRank most readily provides a *ranking for query results*. PageRank's non-principal eigenspaces appear less coherent than its principal eigenspace and are usually associated with significantly smaller eigenvalues. Our qualitative conclusions are supported by more extensive tests and quantitative results that are reported in the 'Discussion' section.

#### Distributions of eigenvalues and prominence values

We study the distributions of the eigenvalues of the different prominence models' connectivity matrices. This analysis can help determine how many different eigenvectors should be used when ranking instance sets (see 'Discussion'). In general, we observe that the eigenvalues produced by sparse models all display similar decay patterns across different queries. PageRank, on the other hand, usually produces one relatively large eigenvalue while the rest of the eigenvalues all have very similar smaller values. We observe these patterns for queries in local and global mode. Our results are reported in the 'Methods' section. We also studied the distribution of prominence values in the *principal *eigenvectors produced by the various prominence models. We observe that for a given prominence model, the properties of the distribution of prominence values within an eigenvector are fairly consistent both across different query terms and across the different eigenvectors of a particular matrix. In general, only a small fraction of prominence values are actually relevant in each eigenspace. However, *a priori *it is unclear which prominence values should be considered significant, since every eigenvector spans the whole query graph and all documents are assigned prominence values in each eigenspace. To address this problem, we model the distribution of low-scoring documents and use that distribution to estimate the significance of outliers. For details see 'Methods'.

#### Eigenspaces and connected components

We examine the correlation between eigenspaces and connected components of the data graph. Previous studies [14,15] observed that the same documents might appear in multiple eigenspaces. Our experiments are in agreement with these observations. For example, the 1977 nodes in the focused subgraph for the search term cancer form 656 connected components (CC), the largest of which contains 264 nodes. The rest of the components are much smaller and the majority of the nodes (1374) are in CC of size 5 or less. The set of top scoring nodes in the first, second, fifth, eight and tenth eigenspaces (computed with the Eigenvector Centrality model) are drawn completely from the largest CC. Some of these objects appear in more than one eigenspace. On the other hand, most of the other connected components in this graph are mapped to specific eigenspaces. For example, the second CC corresponds exactly to the group of top scoring nodes in the third eigenspace. Similarly, the third CC constitutes the the top scoring nodes in the fourth eigenspace. Interestingly, even when overlap is observed (as is the case for the top CC), most of the top scoring nodes in each eigenspace are unique.

We observe similar behavior with the query term ubiquitin. The graph contains 6219 nodes, of which 1811 are in the largest CC. The second largest CC contains only 41 nodes, and 2364 nodes are connected only to one other object. However, in this case the top CC is so large that it completely dominates all top ten eigenspaces. We also observe overlap between these eigenspaces. For example, as was pointed out in section 'Examples', the fifth and sixth eigenspaces rank the same two proteins (UV excision repair protein RAD23 and 26S protease regulatory subunit 7 homolog) at the top, but in reverse order. Interestingly, these two proteins are related by an interaction (docid: 60875934) which is ranked 3rd in the fifth eigenspace but ranked 48th in the sixth eigenspace. Each of these proteins is associated with many interactions, and the immediate structures of the graphs centered at each of these two proteins are somewhat similar with respect to interactions. However, the graph structures are different with respect to nucleic acid sequences as UV excision repair protein RAD23 is linked to a DNA sequence that encodes several other proteins, forming a slightly different community from 26S protease regulatory subunit 7 homolog, whose related DNA sequences do not encode other proteins.

Both examples demonstrate that eigenspaces can overlap. However, each eigenspace is centered on a different subset of objects and the high scoring documents in each one form a different subgraph with a different structure. We postulate that the top-scoring documents in overlapping eigenspaces are indicative of highly non-planar graphs. The prominence models we considered are based on eigenvector analysis and are essentially linear projection methods. These methods fail to faithfully embed complex non-planar subgraphs within a single hyperplane, and therefore we observe multiple non-isomorphic projections of these graphs (for a discussion on embedding algorithms see [19]). We conjecture that such graphs are split between eigenspaces roughly along minimal cuts (i.e. partitions with minimal number of cross edges).

## Discussion

### Evaluating the quality of the results

While each of the prominence models is designed to generate result sets that are sorted based on relevance, it is hard to quantitatively evaluate the quality of these results on a large scale. Clearly, one can provide a few examples demonstrating the properties of a specific system; however, it is difficult to infer general conclusions from a few carefully chosen examples.

An independent measure of quality is especially important when comparing different weighting schemes and evaluating the impact of non-principal eigenspaces (as described in section 'Weighting functions'). Ideally, such a measure would reflect the utility of the results returned by the query. However, utility is a largely subjective matter, especially when considering that Biozon is intended for a wide variety of users with many different interests. Thus, our attempt to define an absolute quality measure on the search results can only be viewed as an attempt to approximate a "consensus" point of view based on the objects themselves. To that end we chose to use the textual information associated with the biological entities, and we validate and assess the performance of a given method by measuring the coherence among the descriptions of instances in the result set and their linked objects. Our intuition is that "good" results are those that not only contain the query term but are also connected to many other objects that contain the query term. Specifically, for each instance *v *in the result set we examine its set of direct neighbors (denoted as the subgraph *G*_*v*_) and count how many of them match the query term (referred to as **consistent neighbors). **Denote by *n*_*v *_the number of direct neighbors of *v *that have definitions (This last condition is required since there are many objects that do not have explicit definitions) and by *m*_*v *_the number of consistent neighbors of *v*. Given the result sets, one can compute the average number of consistent neighbors per query (as a *typical *performance measure), or the total number of consistent neighbors among all queries tested (as an *overall *performance measure). However, a few instances that are connected to many consistent neighbors can dominate and bias these performance measures. Normalized measures, such as the relative measure *m*_*v*_*/n*_*v*_, are not optimal either. Many of the instances are connected to only one other, consistent object. With a relative measure of 1 for these instances, the overall or typical performance will be biased as well by diminishing the contributions of strongly connected instances in the result set. Instead, we propose a probabilistic measure, and for each instance we estimate the significance of observing *m*_*v *_consistent neighbors out of *n*_*v *_neighbors. Formally, given a query term *Q *we estimate the probability that a random object will match the term by *p = N*_*Q*_*/N *where *N*_*Q *_is the total number of objects that match the query term, and *N *is the total number of objects in the Biozon database. (It is possible to obtain better estimates of the parameter *p*. However, the differences are marginal and due to its computational simplicity we chose the latter.) The binomial distribution with parameter *p *can then be used to estimate the probability to observe exactly *m *consistent neighbors out of *n *neighbors, employing the simplifying assumption of independence

Pr⁡(m)=(nm)pm(1−p)n−m.
 MathType@MTEF@5@5@+=feaafiart1ev1aaatCvAUfKttLearuWrP9MDH5MBPbIqV92AaeXatLxBI9gBaebbnrfifHhDYfgasaacH8akY=wiFfYdH8Gipec8Eeeu0xXdbba9frFj0=OqFfea0dXdd9vqai=hGuQ8kuc9pgc9s8qqaq=dirpe0xb9q8qiLsFr0=vr0=vr0dc8meaabaqaciaacaGaaeqabaqabeGadaaakeaacyGGqbaucqGGYbGCcqGGOaakcqWGTbqBcqGGPaqkcqGH9aqpdaqadaabaeqabaGaemOBa4gabaGaemyBa0gaaiaawIcacaGLPaaacqWGWbaCdaahaaWcbeqaaiabd2gaTbaakiabcIcaOiabigdaXiabgkHiTiabdchaWjabcMcaPmaaCaaaleqabaGaemOBa4MaeyOeI0IaemyBa0gaaOGaeiOla4caaa@4480@

The significance (pvalue) of the subgraph *G*_*v *_is estimated by the total probability to observe by chance graphs that are at least as consistent as the observed graph

pvalue(Gv)=Pr⁡(m′>=m)=∑m′=mnPr⁡(m′).
 MathType@MTEF@5@5@+=feaafiart1ev1aaatCvAUfKttLearuWrP9MDH5MBPbIqV92AaeXatLxBI9gBaebbnrfifHhDYfgasaacH8akY=wiFfYdH8Gipec8Eeeu0xXdbba9frFj0=OqFfea0dXdd9vqai=hGuQ8kuc9pgc9s8qqaq=dirpe0xb9q8qiLsFr0=vr0=vr0dc8meaabaqaciaacaGaaeqabaqabeGadaaakeaacqqGWbaCcqqG2bGDcqqGHbqycqqGSbaBcqqG1bqDcqqGLbqzcqGGOaakcqWGhbWrdaWgaaWcbaGaemODayhabeaakiabcMcaPiabg2da9iGbccfaqjabckhaYjabcIcaOiqbd2gaTzaafaGaeyOpa4Jaeyypa0JaemyBa0MaeiykaKIaeyypa0ZaaabCaeaacyGGqbaucqGGYbGCcqGGOaakcuWGTbqBgaqbaiabcMcaPaWcbaGafmyBa0MbauaacqGH9aqpcqWGTbqBaeaacqWGUbGBa0GaeyyeIuoakiabc6caUaaa@52BC@

This measure accounts for both the consistency and the subgraph size. Given the complete result set **R**, the total pvalue is approximated by

pvalue(R)=∏v∈Rpvalue(Gv)
 MathType@MTEF@5@5@+=feaafiart1ev1aaatCvAUfKttLearuWrP9MDH5MBPbIqV92AaeXatLxBI9gBaebbnrfifHhDYfgasaacH8akY=wiFfYdH8Gipec8Eeeu0xXdbba9frFj0=OqFfea0dXdd9vqai=hGuQ8kuc9pgc9s8qqaq=dirpe0xb9q8qiLsFr0=vr0=vr0dc8meaabaqaciaacaGaaeqabaqabeGadaaakeaacqqGWbaCcqqG2bGDcqqGHbqycqqGSbaBcqqG1bqDcqqGLbqzcqGGOaakieqacqWFsbGucqGGPaqkcqGH9aqpdaqeqbqaaiabbchaWjabbAha2jabbggaHjabbYgaSjabbwha1jabbwgaLjabcIcaOiabdEeahnaaBaaaleaacqWG2bGDaeqaaOGaeiykaKcaleaacqWG2bGDcqGHiiIZcqWFsbGuaeqaniabg+Givdaaaa@4BC7@

or with the monotonic log-transformation

Q(R)=−∑v∈Rlog⁡(pvalue(Gv))
 MathType@MTEF@5@5@+=feaafiart1ev1aaatCvAUfKttLearuWrP9MDH5MBPbIqV92AaeXatLxBI9gBaebbnrfifHhDYfgasaacH8akY=wiFfYdH8Gipec8Eeeu0xXdbba9frFj0=OqFfea0dXdd9vqai=hGuQ8kuc9pgc9s8qqaq=dirpe0xb9q8qiLsFr0=vr0=vr0dc8meaabaqaciaacaGaaeqabaqabeGadaaakeaacqWGrbqucqGGOaakieqacqWFsbGucqGGPaqkcqGH9aqpcqGHsisldaaeqbqaaiGbcYgaSjabc+gaVjabcEgaNjabcIcaOiabbchaWjabbAha2jabbggaHjabbYgaSjabbwha1jabbwgaLjabcIcaOiabdEeahnaaBaaaleaacqWG2bGDaeqaaOGaeiykaKIaeiykaKcaleaacqWG2bGDcqGHiiIZcqWFsbGuaeqaniabggHiLdaaaa@4B7C@

This measure, however, is useful only when comparing different sets of results because it does not account for the ordering within the set of results. Our second performance measure evaluates different ranking methods by accounting also for the ordering of objects within the result set. Our method is a variation over the popular ROC measure [20]. However, unlike the typical setting for this measure (which requires labeled data) we have quantitative data with a significance value assigned to each sample. We assume that better models will report the more significant instances first. Therefore, the cumulative area under the curve corresponding to the sorted list of instances (from most significant to least significant) can serve as an overall performance measure. Formally, we are given a set of *N *instances, sorted by ranking function *f*. Denote by **R**(*i*) the set of *i *highest ranked documents. The quality of *f *is estimated by the functional UROC (unsupervised ROC) which we define as

UROCN(R)=∑i=1N∑j=1i−log⁡(pvalue(Gi))=∑i=1NQ(R(i))
 MathType@MTEF@5@5@+=feaafiart1ev1aaatCvAUfKttLearuWrP9MDH5MBPbIqV92AaeXatLxBI9gBaebbnrfifHhDYfgasaacH8akY=wiFfYdH8Gipec8Eeeu0xXdbba9frFj0=OqFfea0dXdd9vqai=hGuQ8kuc9pgc9s8qqaq=dirpe0xb9q8qiLsFr0=vr0=vr0dc8meaabaqaciaacaGaaeqabaqabeGadaaakeaacqWGvbqvcqWGsbGucqWGpbWtcqWGdbWqdaWgaaWcbaGaemOta4eabeaakiabcIcaOGqabiab=jfasjabcMcaPiabg2da9maaqahabaWaaabCaeaacqGHsislcyGGSbaBcqGGVbWBcqGGNbWzcqGGOaakcqqGWbaCcqqG2bGDcqqGHbqycqqGSbaBcqqG1bqDcqqGLbqzcqGGOaakcqWGhbWrdaWgaaWcbaGaemyAaKgabeaakiabcMcaPiabcMcaPaWcbaGaemOAaOMaeyypa0JaeGymaedabaGaemyAaKganiabggHiLdGccqGH9aqpdaaeWbqaaiabdgfarjabcIcaOiab=jfasjabcIcaOiabdMgaPjabcMcaPiabcMcaPaWcbaGaemyAaKMaeyypa0JaeGymaedabaGaemOta4eaniabggHiLdaaleaacqWGPbqAcqGH9aqpcqaIXaqmaeaacqWGobGta0GaeyyeIuoaaaa@6666@

This functional obtains its maximal value when the instances are sorted in increasing order of significance values and obtains its minimal value when instances are sorted in decreasing order of significance.

Therefore, a ranking function that correlates well with ordering by significance values will get a high UROC score. Here we limit N to be 50, and compute *UROC*_50_, assuming that it is not very likely that a user will scan more than the top 50 results. We use this measure to compare different models and variations of prominence models as described next.

Note that the UROC functional induces a certain ordering on instances that we perceive as important, and the best ranking procedure would be a one that is perfectly correlated with this functional. One might argue that instead of using the prominence models, entities should be ranked directly based on these probability-based measures. However, computing these values in real time for an arbitrary query is time consuming and therefore we resort to precomputed indices.

### Assessing the contribution of non-principal eigenspaces

All the models we tested return multiple potential prominence vectors in the form of eigenvectors or vector projections of multidimensional eigenspaces. Each such prominence vector represents a different way of distributing prominence values among the graph nodes. Hence, a given connectivity matrix can produce several potential values for each object.

As the examples in section 'Examples' demonstrated, it is hard to assign a universal meaning to the different eigenspaces. Moreover, not all eigenspaces are equally informative, and entities highly relevant to the query can be distributed across multiple vectors. Furthermore, a document might be included in different groups of inter-related documents and therefore might appear in multiple eigenspaces. Previous studies [14,15] observed that non-principal components may or may not contain useful information. However, no methodological solutions were proposed to handle non-principal eigenspaces.

Here we attempt to quantitatively assess the importance of non-principal eigenspaces and to propose methods for integrating information from multiple prominence vectors. Each method is assessed in terms of its effectiveness in ranking documents in focused subgraphs, using the measures proposed in the previous section. Our goal is to determine if the information stored in the first eigenspace is enough to rank the instances effectively.

#### Weighting functions

We explore several methods for weighting and combining prominence vectors in order to consolidate all information available in the different prominence vectors into a single meaningful ranking system. Given a set of eigenvalues *e*_1_, ..., *e*_*n *_and associated prominence vectors **e_1_**, ..., **e_n _**(where the *i*^*th *^component of each vector corresponds to the *i*^*th*^ document), we define the following weighting functions:

• Principal Eigenspace – A document's final score is its component in the prominence vector corresponding to the principal eigenspace. That is, the *i*^*th *^document's final score is equal to the *i*^*th*^ component of *e*_1_. (this is the approach taken by previous studies on prominence models when ranking web documents).

• Max – A document's final score is the maximum prominence value it receives in any of the eigenspaces. *Max*(*i*) = *max*_*k *= 1...*n*_(**e_k_**(*i*))

• Weighted Max – A document's final score is its maximum prominence value weighted by the corresponding eigenvalue. *WeightedMax*(*i*)* = max*_*k *= 1...*n*_(*e*_*k*_·**e_k_**(*i*))

• Weighted Sum – A document's final score is the sum of the prominence values in each vector weighted by the corresponding eigenvalues. *WeightedSum*(*i*) = ∑k=1n(ek⋅ek(i))
 MathType@MTEF@5@5@+=feaafiart1ev1aaatCvAUfKttLearuWrP9MDH5MBPbIqV92AaeXatLxBI9gBaebbnrfifHhDYfgasaacH8akY=wiFfYdH8Gipec8Eeeu0xXdbba9frFj0=OqFfea0dXdd9vqai=hGuQ8kuc9pgc9s8qqaq=dirpe0xb9q8qiLsFr0=vr0=vr0dc8meaabaqaciaacaGaaeqabaqabeGadaaakeaadaaeWaqaaiabcIcaOiabdwgaLnaaBaaaleaacqWGRbWAaeqaaOGaeyyXICncbeGae8xzau2aaSbaaSqaaiab=TgaRbqabaGccqGGOaakcqWGPbqAcqGGPaqkcqGGPaqkaSqaaiabdUgaRjabg2da9iabigdaXaqaaiabd6gaUbqdcqGHris5aaaa@4043@

#### Comparison of weighting functions

We tested a total of 44 queries with each function. The queries consist of a combination of different target data types, prominence models and query terms. Query terms were chosen purely based on biological interest prior to performance evaluation. Because of the small size of the focused subgraphs (Table 5), we could reliably generate a large number of eigenvectors. The documents in each result set were filtered for the proper data type (i.e. the query data type) and ranked using the four weighting functions over the prominence vectors derived from the top 20 eigenvalues/eigenspaces (for all queries we considered, the information contained in eigenspaces other than the top twenty seem to be marginal). The ordered set of documents was then evaluated using the UROC measure described above, and the results are summarized in Table 1a. While all four functions do well, it appears that the Principal Eigenspace measure produces the best results on average, both in terms of average rank and average ratio.

**Table 1:** Part (a) Comparing weighting functions for combining the contributions of multiple eigenspaces.

	Weighting Function	Max Rank	Min Rank	Average Rank	Max Ratio	Min Ratio	Average Ratio
Part (a)	Principal Eigenspace	1	4	**2.14**	1	0.20	**0.90**
	Weighted Sum	1	4	2.64	1	0.30	0.86
	Weighted Max	2	4	2.84	1	**0.34**	0.85
	Max	1	4	2.37	1	0.32	0.84
Part (b)	Principal Eigenspace	1	4	**1.37**	1	**0.96**	**0.99**
	Weighted Sum	1	4	2.12	1	0.69	0.88
	Weighted Max	3	4	3.37	0.99	0.56	0.84
	Max	2	4	3.12	0.99	0.53	0.85
Part (c)	Principal Eigenspace	1	4	2.8	1	0.20	0.81
	Weighted Sum	2	4	2.9	1	0.37	0.83
	Weighted Max	2	3	2.3	1	0.37	**0.84**
	Max	1	4	**2**	1	**0.49**	0.83

To evaluate each weighting function *f *we ran a total of 44 queries. Each query is a distinct combination of (search term, search type, ranking method), for example (ubiquitin, protein, PageRank). For each query we analyzed the focused subgraph and computed the top 20 eigenvectors. The contributions of their derived prominence vectors were weighted using the function *f *by assigning a total score *f*(*d*) to each document *d*. The documents were sorted based on their total score, and the quality of top fifty documents was assessed in terms of the *UROC *measure (see text). The different weighting functions were then ranked based on their performance on each query. The minimum, maximum and average rankings for each weighting function over these queries are listed. Furthermore, the ratio of the given function's quality to the maximum quality achieved in that query is also computed and the minimum, maximum and average of this value are computed over the 44 queries. Part (b) Comparison of weighting functions with the PageRank model. Results are reported based on 8 distinct queries in focused subgraph mode. With this model, the Principal Eigenspace clearly dominates performance. Part (c) Comparison of weighting functions with the Hubs & Authorities model. Results are reported based on 10 distinct queries in focused subgraph mode. Unlike PageRank, this model performs better when information from non-principal eigenspaces is included. The best weighting function is Max (see Section).

Analyzing the results by prominence methods reveals an interesting trend that supports our qualitative results reported in section 'Examples'. Table 1b shows the results for the four different weighting functions when considering only the PageRank method. Here, ranking documents by the Principal Eigenspace dominates performance in all aspects. This indicates that in general the non-principal eigenvectors produced using the PageRank method are much less informative than the principal eigenvector. On the other hand, when considering only the Hubs & Authorities model (Table 1c) the Principal Eigenspace method does significantly worse than the others, which indicates that useful information can be found in Hubs & Authorities's non-principal eigenvectors. The differences are even more significant for Eigenvector Centrality (results not shown).

### Comparison of prominence models

**Figure 2:**
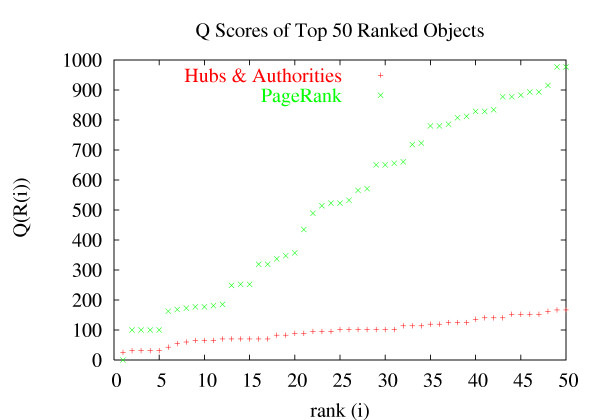
***UROC *curves for PageRankand Hubs & Authorities**. The result sets were produced from querying proteins using the search term ubiquitin, in global mode. The *UROC *score is the area under the curve.

Practical considerations motivated us to look further into global methods (see section 'Search strategies' in 'Results'), since computing the focused subgraphs is a relatively slow process. Global methods provide us with precomputed scores that can be used to rank documents returned by arbitrary queries on the fly. However, global methods are bounded by other computational issues, and generating more than just the principal eigenspace can be very expensive. Encouraged by the relative success of the PageRank method with its principal eigenspace, we ran a second set of experiments to re-evaluate which prominence method is the most effective in *global mode *when only the principal eigenspace is used. Detailed results are given in Table 2, and example *UROC *graphs are shown in Fig. 2. The results are consistent and suggest that PageRank is the most successful method under this setup.

**Table 2:** Comparison of prominence models on the global Biozon graph.

Query Term	Query Type	Prominence Model	Average No. Neighbors	Average Consistent Neighbors	Average Ratio	*Q*(**R**)	*UROC*(**R**)
		Hubs & Authorities	4.38	0.6	0.13	167	4879
ubiquitin	protein	Eigenvector Centrality	4.38	0.6	0.13	167	4879
		Katz's Status	3.82	0.48	0.12	135	3436
		PageRank	12.18	3.56	0.27	977	**26021**
		Hubs & Authorities	1.19	0.79	0.48	357	10086
stromelysin	protein	Eigenvector Centrality	1.19	0.79	0.48	357	9912
		Katz's Status	1.19	0.79	0.48	357	10061
		PageRank	1.19	0.79	0.48	357	**11593**
		Hubs & Authorities	5.22	1.08	0.25	317	6137
cancer	protein	Eigenvector Centrality	5.20	0.9	0.21	245	5346
		Katz's Status	4.84	0.64	0.19	170	4915
		PageRank	6.68	1.8	0.30	535	**16082**
		Hubs & Authorities	1.26	0.5	0.46	166	4628
cancer	nucleic	Eigenvector Centrality	1.26	0.46	0.45	151	**4635**
		Katz's Status	1.08	0.44	0.44	146	4529
		PageRank	1.60	0.52	0.39	167	4633
		Hubs & Authorities	1.1	0.67	0.58	223	4520
autoimmune	protein	Eigenvector Centrality	1.1	0.67	0.58	223	4520
		Katz's Status	1.1	0.67	0.58	223	4520
		PageRank	1.1	0.67	0.58	223	**4582**
		Hubs & Authorities	0.98	0.4	0.4	226	4559
autoimmune	nucleic	Eigenvector Centrality	0.98	0.4	0.4	226	5430
		Katz's Status	0.98	0.4	0.4	226	4401
		PageRank	0.98	0.4	0.4	226	**7659**

Only the principal eigenspace is used. For each query consisting of (query term, query type, prominence model) we report the following results over the set R of the top 50 documents returned by that query: The average number of neighbors per document in that set, the average number of consistent neighbors, the average ratio of these numbers, the quality of results set *Q*(R), and the most informative measure *UROC*(R).

**Figure 3:**
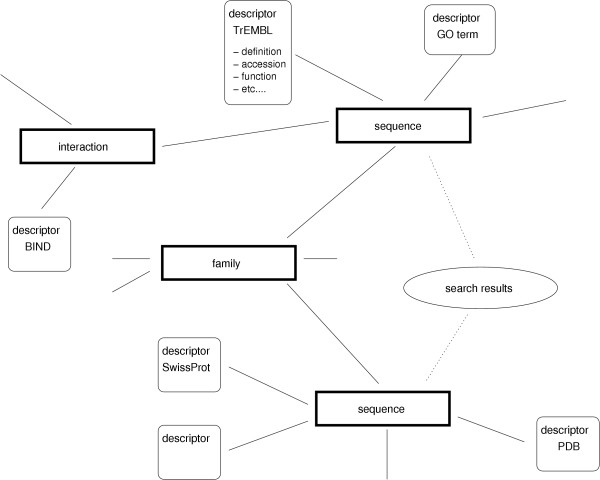
**A subgraph of objects and descriptors**. Objects, descriptors, and the relations between them form a typical subset of the Biozon data graph. Search results return sets of objects, from which the graph can be navigated to see the object's context.

### The effect of normalization

We conjecture that the observed differences in performance between the prominence models is an artifact of matrix normalization. The PageRank model uses a connectivity matrix in which every row is normalized to sum to 1. This means a node splits its rank evenly among its neighbors. The other three models do not normalize their connectivity matrices. In these models, a node confers its whole rank upon each of its neighbors. For example, consider a graph containing (among other things) a large protein family with *n *related proteins. Suppose each protein is only connected to the family. With a normalized matrix, each of the proteins confers its whole rank to the family, but the family only confers 1/*n *of its rank back to the proteins. Consequently, although many proteins contribute to the family to give it a high rank, the family splits this rank among its proteins and so the individual proteins' ranks remain low. Thus PageRank emphasizes the family without emphasizing its members. On the other hand, with an unnormalized matrix, the protein family confers its whole authority upon each of its members. Thus if the protein family is highly ranked, its members will also be highly ranked. Hence an unnormalized matrix method will tend to co-emphasize protein families and their members.

We tested the effect of normalization on the performance of the prominence models. The results are summarized in Table 3. As the table shows, normalization can have a drastic impact on the results, and performance almost always improves with normalized connectivity matrices. However, note that PageRank still performs better.

**Table 3:** The effect of normalization on the ranking.

Query Term	Query Type	Prominence Model	Average No. Neighbors	Average Consistent Neighbors	Average Ratio	*Q*(**R**)	*UROC *(**R**)
		Hubs & Authorities	4.38	0.6	0.13	167	4879
		Hubs & Authorities(N)	7.64	2.58	0.52	705	23809
ubiquitin	protein	Eigenvector Centrality	4.38	0.6	0.13	167	4879
		Eigenvector Centrality(N)	1.04	0.9	0.88	307	7871
		PageRank	12.18	3.56	0.27	977	**26021**
		Hubs & Authorities	1.19	0.79	0.48	357	10086
		Hubs & Authorities(N)	1.19	0.79	0.48	357	10112
stromelysin	protein	Eigenvector Centrality	1.19	0.79	0.48	357	9912
		Eigenvector Centrality(N)	1.19	0.79	0.48	357	9057
		PageRank	1.19	0.79	0.48	357	**11593**
		Hubs & Authorities	5.22	1.08	0.25	317	6137
		Hubs & Authorities(N)	3.62	1.2	0.67	372	11947
cancer	protein	Eigenvector Centrality	5.2	0.9	0.21	245	5346
		Eigenvector Centrality(N)	1.24	0.98	0.86	321	8137
		PageRank	6.68	1.8	0.30	535	**16082**
		Hubs & Authorities	1.1	0.67	0.58	223	4520
		Hubs & Authorities(H)	1.1	0.67	0.58	223	4345
autoimmune	protein	Eigenvector Centrality	1.1	0.67	0.58	223	4520
		Eigenvector Centrality(H)	1.1	0.67	0.58	223	**4830**
		PageRank	1.1	0.67	0.58	223	4582

For each model we repeated the computations with the unnormalized and the normalized (N) matrices. See Table 2 for details.

### Local vs. global methods

We compare the performance of the PageRank model using focused subgraph, global and extended global modes (section 'Search strategies'). We observe that the global mode sometimes outperforms focused subgraph mode with respect to the *UROC*_50 _measure (see Table 4). This is possibly due to "edges effects": objects that are excluded from the focused graph are considered in global mode and can increase the prominence values of their neighbors which, in turn, increase the prominence values of the objects relevant to the query term. However the global mode is outperformed when it produces less than 50 results. The extended global mode improves over the global mode and seems to produce results that are comparable to the best mode for each query. These results suggest that the extended global mode is a good compromise between sensitivity and speed. Interestingly, the top result returned for the stromelysin query in extended global mode is, in fact, a protein whose definition does not contain the search term. This protein, interstitial collagenase precursor (docid 884427), is similar to several other stromelysin proteins and is encoded by a stromelysin DNA gene. For comparison, we also provide the baseline performance. This was evaluated by picking 50 random documents from the subgraph and computing UROC based on the random sampling order (the procedure was repeated 50 times and the results were averaged).

**Table 4:** Performance evaluation: focused subgraph vs. global vs. extended global.

Query Term	Query Type	Graph	Number of Results	Average No. Neighbors	Average Consistent Neighbors	Average Ratio	*Q*(**R**)	*UROC *(**R**)
ubiquitin	protein	Baseline	50	1.92	1.0	0.64	329	8278
		Focused	50	11.72	2.9	0.20	771	18516
		Global	50	12.18	3.56	0.27	977	26021
		Global-ext	50	12.18	3.56	0.27	977	25967
stromelysin	protein	Baseline	46	1.30	0.80	0.49	387	9038
		Focused	46	1.30	0.80	0.49	387	13180
		Global	43	1.19	0.79	0.48	357	11593
		Global-ext	46	1.30	0.80	0.49	387	13045
cancer	protein	Baseline	50	2.01	1.03	0.73	323	8202
		Focused	50	6.72	1.74	0.28	516	14901
		Global	50	6.68	1.8	0.30	535	16082
		Global-ext	50	6.72	1.8	0.30	534	16010
autoimmune	protein	Baseline	50	1.92	0.84	0.60	455	11512
		Focused	50	2.33	1.02	0.73	530	14330
		Global	30	1.1	0.67	0.58	223	4582
		Global-ext	50	2.24	0.98	0.70	530	14054

All results were generated with the PageRank prominence model, using the Principal Eigenspace scoring function. In the cases of autoimmune and stromelysin, the global method returns less than 50 documents. The extended global mode reports more than 50 documents, which explains the larger overall quality.

**Table 5:** Makeup of the focused subgraphs for selected queries.

Query Term	Query Type	Proteins	Nucleic Acid Sequences	Protein Families	Structures	Interactions	Pathways	Total	Connected
autoimmune	protein	58	324	1	0	4	0	387	111
	nucleic	58	369	0	0	0	0	427	150
cancer	protein	830	37023	7	5	109	0	37974	1977
	nucleic	829	37300	0	1	1	0	38131	1920
stromelysin	protein	46	566	3	2	0	0	617	76
ubiquitin	protein	2372	28820	9	25	720	1	31947	6219

For each query we list the number of instances of each Biozon data type included in the corresponding focused subgraph. The query types we experimented with are protein sequence and nucleic sequence. One might be surprised by the fact that a structure is included in the focused subgraph for the stromelysin-nucleic search since protein structures are not directly related to nucleic acid sequences. Such nodes are included by definition since they have the search term; however, they are ignored (along with all other nodes that do not have neighbors) in the subsequent eigencalculations and are assigned prominence values of 0. The 'Total' column indicates the number of nodes included in the focused subgraph, while the last column ('Connected') lists the number of nodes of the focused subgraph with at least one neighbor in the subgraph. Only nodes which have neighbors are included in our computations.

### Variations

We tested several other variations of prominence models that exploit the structure of the Biozon graph and the different data and relation types. Our first experiment used directed graphs where edges are directed towards a specific data type (forced directed graph). The main advantage of this method is that it produces a clear and sensible distinction between hubs and authorities, unlike undirected graphs. We also attempted to incorporate the similarity relation into our graphs to help characterize new and unannotated objects. We found that in some cases similarity data caused an immediate and significant improvement in result set quality. Unfortunately, these improvements were not observed consistently across multiple queries, indicating that the problems of redundancy and localization (as discussed in subsection 'Variations on prominence models' of the 'Methods' section) need to be considered. Finally, to account for possible discrepancies in significance among biological relations, we weighted the entries of the adjacency matrices based on the type of the relation. While in some cases edge weighting clearly improves the results, in others it does not, and further study is necessary to converge to a stable and consistent weighting scheme. Our experiments are reported in more detail in the 'Methods' section.

## Conclusion

In this paper we present a system that ranks biological entities returned as results from querying heterogeneous biological data. We view important or interesting instances in the result sets as those that are linked to many other important entities. Since these instances are associated with myriad of biological knowledge (through their relations to other biological entities) they can serve as a useful entry point to researchers who would like to study similar systems. To identify these instances we analyze the intricate link structure of Biozon by applying spectral methods. We test several popular prominence models, variations of these models, different query modes, and different scoring functions. To evaluate the quality and effectiveness of these models we propose an objective probabilistic measure, *UROC*, that accounts for both the structure of the Biozon graph and the textual information contained therein. This measure quantifies the thematic unity within instance subgraphs, directed at detecting what we call "hubs of knowledge".

We examine several issues with prominence models that have not been quantitatively addressed so far. We evaluate the utility of information contained in non-principal eigenspaces as well as different ways to incorporate this information into our prominence models. Our tests indicated that with a certain family of prominence models (including Eigenvector Centrality, Hubs & Authorities and Katz's Status) the information is distributed across multiple eigenspaces and that the Max weighting function is the most effective approach for ranking documents with these models. On the other hand, non-principal eigenspaces seem to contain little information when using the PageRank model. For PageRank, the most effective ranking is produced by relying solely on prominence values in the principal eigenspace. The differences between the PageRank model and the other three models are attributed to two factors. First, PageRank includes a prior matrix that essentially connects all documents. Therefore, PageRank analyzes a completely connected graph while all other models analyze sparse graphs. Second, the rows of PageRank's connectivity matrix are normalized. Indeed, the performance of the other models improved when their connectivity matrices were normalized in a similar manner. Furthermore, we observe that the sparse models are most effective for producing hubs of knowledge while PageRank is the most effective model for ranking query results.

We also compare different search strategies. While the local method can bring forward documents that do not match the query term (the function of these documents is inferred from their broader graph context, through relations to other, better characterized objects), it is computationally prohibitive in real-time. In comparison, global methods rely on pre-computed prominence values and no additional preprocessing is required during query time execution. We also introduced the extended global method that combines the benefits of both the focused subgraph method and the global method, thus allowing fast propagation of information to uncharacterized or unstudied objects.

Practical considerations promoted the use of global methods for a real-time ranking system. However, due to the size of the global connectivity matrix, this choice precludes us from computing many eigenspaces. Since PageRank's principal eigenspace tends to incorporate information from the other methods' non-principal eigenspaces, we conclude that the PageRank model with the principal eigenspace scoring function is the most feasible system for ranking Biozon query results. Indeed, our experiments have shown that PageRank produces the best results given these restrictions. Moreover, PageRank's performance under these conditions is comparable to the best performance observed from any combination of model and scoring function used in local or global mode. The PageRank model has other interesting aspects. For example, the inclusion of the matrix **E **allows one to explore various types of prior knowledge.

One advantage that Biozon has over the World Wide Web is that for the most part Biozon data is highly reliable. While web search engines such as Google have to deal with dynamic data of questionable quality, almost all relations in the Biozon database are well established relations that are derived from high quality observations and measurements. Therefore, our models do not have to address directly issues such as noise and "dead links". There are some exceptions. For example, high-throughput interaction data that is generated using the yeast two-hybrid system tends to contain many false positives. However, these relations constitute a small fraction of the Biozon data graph, and our tests indicate that their presence does not change the ranking significantly.

The heterogeneous data sets reveal other interesting properties and challenges. For example, upon testing the effect of similarity data on performance we observed that similarity relations can be useful, but they can also lead to mediocre performance because of problems of redundancy and localization. Thus, similarity data should be considered with the proper normalization. It should be noted that much of the similarity information already exists in another form. For instance, proteins are often classified to families based on similarity data and thus forms paths (of length two) between similar proteins in the Biozon data graph. Like our tests with similarity data, our tests with edge weighting open another direction that requires further analysis. Here, future work includes exploring more robust weighting and normalization schemes. Another type of information that is currently ignored is the context (such as the species).

Variations that will focus on organism-specific subnetworks will be another topic of future research, as well as the integration of information retrieval techniques and other variations of prominence models. Of special interest are non-linear projection methods that can handle large, non-planar graphs.

The application of all prominence models we tested involves non-trivial issues, and further improvements to a real-time database query system require additional study of the underlying data graph. In particular, it is desirable to precompute as much data as possible to minimize the amount of processing required for each individual query. Among the issues that one has to consider is the overlap between different eigenspaces and the meaning of different eigenspaces. One possibility is to utilize the causal structure and the connectivity within the graph as analysis can be carried out separately on each connected component. To this end, we have isolated the connected components of the Biozon data graph and are studying their properties.

Finally, we should note that although the examples we used in the paper are single term queries, Biozon has the capability of ranking any query the user may execute. This includes multiple term queries and queries involving relations among multiple data types. The current ranking system, based on the PageRank prominence model, was integrated into the Biozon database and is available online at .

## Methods

### The Biozon database and data graph

Biozon is a system that consolidates multiple biological databases consisting of a variety of heterogeneous data types (such as DNA sequences, proteins, interactions and cellular pathways) into a single extensive schema that is logically represented as a large **data graph **Σ. Each node represents some datum, and an edge between two nodes represents a relationship between them. Formally, Σ = (**D**, **R**) where **D **= {*d*_1_... *d*_*n*_} is the set of all nodes (documents) in the graph and **R **= {*r*_1_...*r*_*m*_} ⊂ **D **× **D **is the set of all edges (relations) in the graph. The data is warehoused locally so the fundamental biological objects represented are *non-redundant *even though data within and between their originating sources overlap. The Biozon resource is available online at .

Much of the data in Biozon is gleaned from publicly available databases such as SwissProt, PDB, GenBank, BIND, KEGG, and more (referred to as **source data**). These sources provide the fundamental biological objects in Biozon, many of the relationships that exist between objects, and the annotation that makes it possible for humans to understand this data. Biozon augments the source data with **derived data. **Derived data encompasses any data produced as the result of a computation or operation over some set of existing data in Biozon, and is unavailable elsewhere. Currently, derived data available in Biozon consists of similarity relations between protein sequences and protein structures, domain structure of proteins, and more. Derived data is a substantial part of the Biozon database.

One main advantage of Biozon is that it allows users to form complex queries. For example, one can search for all proteins which have solved structures and are members of enzyme families integral to a particular pathway (note this query spans four different data types: protein sequences, protein structures, protein families, and pathways). Similarly, one can search for all structures of proteins that are involved in known interactions and so on. In addition to addressing the problem of data integration and unification from multiple resources, complex queries enable sophisticated data manipulation. For more information see [21].

#### Documents

Each biological entity (be it source data or derived data) is represented as a set of documents. In general, documents are divided into a hierarchy of categories. At the top of the hierarchy we distinguish between **objects **and **descriptors. **Objects are basic biological entities. An object is either a physical entity (i.e. protein sequence, protein structure), or a group of physical entities (i.e. interaction, protein family, pathway) A Descriptor, as the name implies, is any piece of knowledge associated with a physical entity, be it a narrative description, a feature, or a measurement (such as expression data). Each document is represented as a node in the Biozon data graph.

For example, a SwissProt record will be mapped to a pair of documents: an *object *document that corresponds to the physical amino acid sequence and a SwissProt *descriptor *document that contains all the expert knowledge stored in SwissProt associated with that protein. A relation is also defined between the two documents to indicate that the descriptor *describes *the object. If the same protein exists in PIR [2], then the PIR entry will be mapped to the same *object *document. A new descriptor, containing the knowledge stored in PIR, will be introduced, and a relation between this new descriptor and the object will be created.

#### Relations

Relations are the edges that connect the documents of the data graph. Relations consist of pointers to a referring document and a referred document. The types of relations classified in the Biozon database include 'member of,' 'manifests as,' 'describes,' 'encodes,' 'similar,' 'contains,' 'expresses' and 'comprised of.' Each relation implies a particular directional meaning, in particular specifying the types of documents related. For example, the 'describes' relation represents the relation between a referring descriptor and a referred object. Likewise, the 'encodes' relation represents the relation between a referring nucleic acid sequence and the referred amino acid sequence that it encodes. By definition, the reverse edges also exist (e.g. 'described by' or 'encoded by'), however only one of the edges is physically stored in the database. The default direction is chosen to be the active voice name of the relation.

#### Directed vs. undirected graph

The edges of the Biozon data graph correspond to the relations between the different entities in the graph. We consider two variations over this graph: the directed and the undirected. In the directed one, there is an directed edge from object *a *to object *b *in the graph if and only if object *a *refers to object *b *in the appropriate relationship in Biozon, as defined by the active voice name of the relation. The undirected graph is simply the directed subgraph after adding in the reverse edges as well.

Although in some cases the direction of the relationship might imply causality (e.g. DNA sequence encodes for a protein), most relationships are such that both directions are biologically valid and no particular direction is more significant than the other. Moreover, for most practical purposes it is easier to work with undirected matrices, since they are symmetric. Therefore our default setup throughout this paper is of undirected graphs. We investigate the relationship between the properties of directed and undirected graphs in section 'Forced directed graphs'.

#### Data types

**Figure 4:**
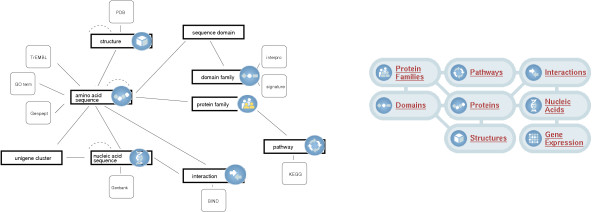
**The Biozon schema and datagraph**. Left: partial high level description of the actual physical schema (see [21] for a detailed description of the schema). Right: the set of main *objects *in Biozon and their relations (some entities are omitted for clarity).

In this study we consider the following data types: nucleic acid sequences, protein sequences, protein structures, enzyme families, interactions, and pathways as well as the relationships among these types (including similarity relations). The global data graph and a partial overview of the Biozon schema are displayed in Fig. 4. This graph, at the instance level, is the subject of our analysis of prominence models. While the descriptor documents describe the biological entities that are relevant to a particular query, they are not considered when analyzing the Biozon link structure and assigning prominence to nodes. This is because the information that is stored in biological databases is often redundant, and many biological entities are reported in multiple databases with almost identical descriptors. In this work, the prominence of an object is defined based on the set of other *biological entities *that are connected to it.

### Prominence models

All the prominence models we consider are based on the idea that a node is prominent if it is connected to other prominent nodes. We study four main methods for assigning prominence values to nodes, as described next. Given a query, the analysis starts by defining the graph (or subgraph) of relevant documents and their **adjacency matrix A. **Each method differently characterizes the connectivity within the data graph to derive a **connectivity matrix B **from the adjacency matrix. The spectral properties of the connectivity matrix are then analyzed by computing its eigenvectors. Each eigenvector is considered to be a possible assignment of prominence values to documents, where node *u *is assigned a prominence value equal to the *u*^*th *^component of the eigenvector. The highest scoring nodes in the principal vector(s) are returned as potential significant matches.

#### Eigenvector centrality

**Figure 5:**
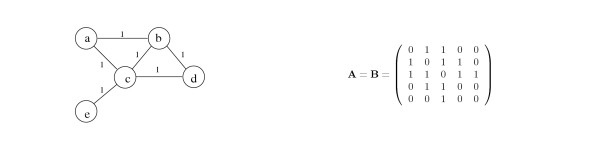
A sample data graph and the corresponding connectivity matrix **B **with the Eigenvector Centrality model. The connectivity matrix is derived directly from the adjacency matrix of the graph.

The Eigenvector Centrality model focuses on direct edges between nodes (see Fig. 5) and defines the prominence of a node to be proportional to the sum of the prominence values of all nodes connected to it. Let **p **be a vector of prominences and denote the prominence of node *v *by *p*(*v*), then

p(v)∼∑(u,v)∈Rp(u)
 MathType@MTEF@5@5@+=feaafiart1ev1aaatCvAUfKttLearuWrP9MDH5MBPbIqV92AaeXatLxBI9gBaebbnrfifHhDYfgasaacH8akY=wiFfYdH8Gipec8Eeeu0xXdbba9frFj0=OqFfea0dXdd9vqai=hGuQ8kuc9pgc9s8qqaq=dirpe0xb9q8qiLsFr0=vr0=vr0dc8meaabaqaciaacaGaaeqabaqabeGadaaakeaacqWGWbaCcqGGOaakcqWG2bGDcqGGPaqkcqWI8iIodaaeqbqaaiabdchaWjabcIcaOiabdwha1jabcMcaPaWcbaGaeiikaGIaemyDauNaeiilaWIaemODayNaeiykaKIaeyicI4mcbeGae8NuaifabeqdcqGHris5aaaa@4147@

Let **A **be the graph's adjacency matrix and **A' **be the transpose of **A**. Then **p **should satisfy **p **~ **A'p **and the solutions of this equation are the eigenvectors of **A'**. Thus we define the connectivity matrix **B **to be **A'**. For undirected graphs **A' **= **A**, and the connectivity matrix **B **is equal to to the adjacency matrix.

#### Hubs and authorities

Hubs & Authorities [14] extends Eigenvector Centrality by differentiating nodes that *have useful information ***(authorities) **from nodes that *link to nodes with useful information ***(hubs). **A node's authority score is proportional to the sum of the hub scores of nodes that link to it, and a node's hub score is proportional to the sum of the authority scores of the nodes it links to.

**Figure 6:**
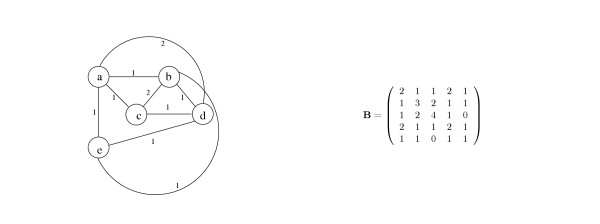
Given the data graph from Fig. 5, Hubs & Authorities analyzes the connectivity matrix **B **where the (*i*, *j*) entry corresponds to the number of length 2 paths between nodes *i *and *j*, as depicted in the graph on the left. Note that with undirected graphs the diagonal elements (*i,i*) of **B **correspond to the number of edges occurring at node *i *in the original data graph.

Let **a **be a vector of authority scores and **h **be the corresponding vector of hub scores. Then **a ~ A'h **and **h ~ Aa**. Hence, **a ~ A'Aa **and **h ~ AA'h**. Thus, in the Hubs & Authorities model, two connectivity matrices are defined and analyzed: **B_1 _**= **A'A **and **B_2 _**= **AA' **where the eigenvectors of **B_1 _**correspond to authority scores and the eigenvectors of **B_2 _**correspond to hub scores. Since **A'A **and **AA' **are always symmetric and positive semi-definite, hubs and authorities vectors are always well-defined in the sense that the power iteration method (see section 'Computing prominence vectors' below) always converges. Note that for an undirected graph **A **is symmetric and therefore **A'A **= **AA' **= **A**^2^. Thus in the undirected case, hub scores and authority scores collapse into a single score. Note also that **A**^2^(*i*, *j*) is the number of paths of length two linking nodes *i *and *j*. Therefore undirected Hubs & Authorities is equivalent to Eigenvector Centrality applied to a new graph in which *i *and *j *are considered adjacent if they are connected by a path of length two in the original graph, and the (*i*, *j*) edge in the new graph is weighted by the number of length two paths connecting *i *and *j *in the original graph (see Fig. 6). In the directed case, the weight of edge (*i*, *j*) in **AA' **(the "coupling" matrix in bibliographies) is the number of nodes that are "co-cited" by both *i *and *j*. The weight of edge (*i*, *j*) in **A'A **(the "co-citation" matrix in bibliographies) is the number of nodes that "co-cite" both *i *and *j*. However, in both cases (of directed and undirected graphs) length one paths in the original graph are not explicitly used, thus possibly eliminating useful information. In this view, we suggest a new model that explicitly utilizes information provided by length one, two, and three paths (see 'Hybrid Katz's Status' below).

#### PageRank

**Figure 7:**
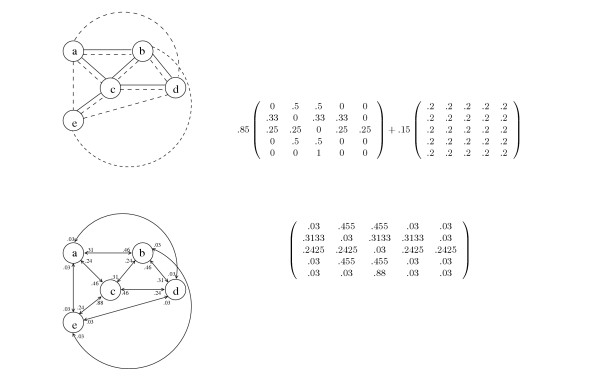
PageRank normalizes the adjacency matrix and adds a prior matrix (top), to produces the connectivity matrix (bottom). Dashed edges (top graph) correspond to a random walk through the document space. Although the original data graph (Fig. 5) is undirected, the edge weights in the PageRank connectivity matrix can differ, depending on the direction, due to normalization.

PageRank [13] uses a probabilistic model to assign prominence values to nodes. With probability *α*, the prominence of node *v *is transferred to a node *u *that *v *points to, where *u *is chosen uniformly at random from the nodes that *v *points to. With probability 1 - *α *the prominence of node *v *is transferred to a node *u *in the graph, chosen randomly with probability *E*(*u*) where **E **is a prior probability distribution over the nodes of the graph. The prior can be interpreted as a random walk through the graph (by a random "graph surfer"), where the probability of a random restart is 1 – *α*, and prominence values reflect the probability that a given node will be visited on such a walk. Thus, PageRank corresponds to Eigenvector Centrality applied to the connectivity matrix **B **= *α*·**A' **+ (1 - *α*)·**E**·**1' **where **A **is the graph's adjacency matrix with rows normalized to sum to 1, and **1 **is a vector of 1's. Note that due to the introduction of the prior matrix, **B **is always dense (in the sense that every node contributes to the prominence value of every other node) and PageRank practically analyzes a completely connected graph (see Fig. 7). In contrast, all other models analyze sparse graphs where only adjacent or nearly adjacent nodes directly contribute to the prominence value of a given object.

#### Hybrid katz's status

A simple measure of prominence that was suggested in [22] is to assign a node's prominence value in proportion to the node's in-degree. However, this measure has some disadvantages compared to those already discussed. Kleinberg [14] discusses the shortcomings of in-degree based ranking; specifically, such rankings tend to emphasize universally popular entities which lack thematic unity. Our Katz's Status model is based on the early work by Katz [23], modeling status in social networks. We generalize the prominence by in-degree idea by assigning a node's prominence in proportion to the number of paths of arbitrary length ending at the node, where long paths are penalized by a decay factor. Note that for adjacency matrix **A **(directed or undirected), **A**^*k*^(*i*, *j*) is the number of *i → j *paths of length *k*. For undirected **A**, we have described spectral methods corresponding to *k *= 1 (Eigenvector Centrality) and *k *= 2 (Hubs & Authorities). More generally, we can define the prominence of node *v*, *p*(*v*) as follows:

p(v)∼∑uw1n1(u,v)p(u)+∑uw2n2(u,v)p(u)+∑uw3n3(u,v)p(u)+…
 MathType@MTEF@5@5@+=feaafiart1ev1aaatCvAUfKttLearuWrP9MDH5MBPbIqV92AaeXatLxBI9gBaebbnrfifHhDYfgasaacH8akY=wiFfYdH8Gipec8Eeeu0xXdbba9frFj0=OqFfea0dXdd9vqai=hGuQ8kuc9pgc9s8qqaq=dirpe0xb9q8qiLsFr0=vr0=vr0dc8meaabaqaciaacaGaaeqabaqabeGadaaakeaacqWGWbaCcqGGOaakcqWG2bGDcqGGPaqkcqWI8iIodaaeqbqaaiabdEha3naaBaaaleaacqaIXaqmaeqaaOGaemOBa42aaSbaaSqaaiabigdaXaqabaGccqGGOaakcqWG1bqDcqGGSaalcqWG2bGDcqGGPaqkcqWGWbaCcqGGOaakcqWG1bqDcqGGPaqkaSqaaiabdwha1bqab0GaeyyeIuoakiabgUcaRmaaqafabaGaem4DaC3aaSbaaSqaaiabikdaYaqabaGccqWGUbGBdaWgaaWcbaGaeGOmaidabeaakiabcIcaOiabdwha1jabcYcaSiabdAha2jabcMcaPiabdchaWjabcIcaOiabdwha1jabcMcaPaWcbaGaemyDauhabeqdcqGHris5aOGaey4kaSYaaabuaeaacqWG3bWDdaWgaaWcbaGaeG4mamdabeaakiabd6gaUnaaBaaaleaacqaIZaWmaeqaaOGaeiikaGIaemyDauNaeiilaWIaemODayNaeiykaKIaemiCaaNaeiikaGIaemyDauNaeiykaKIaey4kaSIaeSOjGSealeaacqWG1bqDaeqaniabggHiLdaaaa@6E9F@

where *n*_*k*_(*u*, *v*) is the number of paths of length *k *between *u *and *v *and *w*_*k *_is the weight of a path of length *k*. Thus, Katz's Status essentially builds a connectivity matrix **B **where **B**(*i*, *j*) is a score based on all paths from *i *to *j*. Fixing the maximal path length *k *and letting

B=∑i=1kwiAi
 MathType@MTEF@5@5@+=feaafiart1ev1aaatCvAUfKttLearuWrP9MDH5MBPbIqV92AaeXatLxBI9gBaebbnrfifHhDYfgasaacH8akY=wiFfYdH8Gipec8Eeeu0xXdbba9frFj0=OqFfea0dXdd9vqai=hGuQ8kuc9pgc9s8qqaq=dirpe0xb9q8qiLsFr0=vr0=vr0dc8meaabaqaciaacaGaaeqabaqabeGadaaakeaaieqacqWFcbGqcqGH9aqpdaaeWbqaaiabdEha3naaBaaaleaacqWGPbqAaeqaaOGae8xqae0aaWbaaSqabeaacqWGPbqAaaaabaGaemyAaKMaeyypa0JaeGymaedabaGaem4AaSganiabggHiLdaaaa@3B43@

we can represent this scheme as matrix multiplication **p ~ Bp **which corresponds to Eigenvector Centrality applied to **B**. Here we set *k = *3, since in the Biozon data graph (see Fig. 4b) most data types can reach any other data type by a path of length 3 at most, and define *w*_1 _= 1, *w*_2 _= 1/16, and *w*_3 _= 1/64. To avoid situations in which cycles undesirably increase the prominence of a node we focus just on simple paths. A simple *i *→ *j *path is a path that does not visit the same vertex twice. Computing the number of simple paths of length > 3 is intractable in general, but is relatively easy for paths of length ≤ 3.

### Computing prominence vectors

In the prominence models we considered, prominence values are associated with the eigenvectors of the corresponding connectivity matrices. As such, any method for finding eigenvectors will suffice. In the particular case of Hubs & Authorities, we use singular value decomposition [24] to simultaneously generate the eigenvalues and eigenvectors of both **A'A **and **AA**. This technique produces an orthonormal basis of each eigenspace without explicitly computing **AA' **or **A'A**.

**Figure 8:**
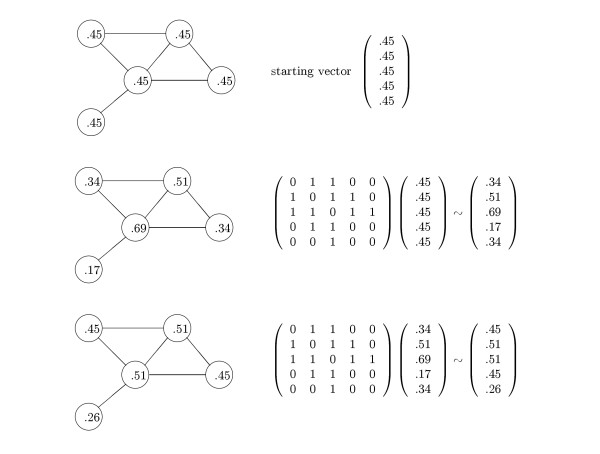
**Computing prominence vectors with the power iteration method**. Starting from a uniform set of values, the prominence vector is iteratively redefined by multiplying it by the adjacency matrix.

One can also use the fast power iteration method [25] to compute the *principal *eigenvalue/eigenvector pair of the connectivity matrix **B **by repeatedly multiplying **B **to some starting vector until convergence (see Fig. 8). Due to computational constraints, the power iteration is the only feasible method to compute eigenvectors of large, dense matrices. However, the power iteration most readily computes a matrix's principal eigenvector and a more elaborate manipulation is required to compute non-principal eigenvectors. The computation of prominence vectors is complicated by multidimensional eigenspaces. For eigenvalues with multiplicity greater than 1, the eigenvectors returned by standard linear algebra packages may be arbitrary and ambiguous. The power iteration will also generate suboptimal results in this setting: if all eigenvalues are positive, it will converge to an arbitrary vector dependent on the starting vector, and if the eigenvalues are of opposite signs (± λ), it will bounce back and forth between two vectors. Note that by definition, any vector in a multidimensional eigenspace is a prominence vector. Therefore, the selection of any single vector of prominence values from the space will be completely arbitrary. Moreover, much of the information about a multidimensional eigenspace is lost when only one vector is selected. Therefore, we take the prominence of an object *i *in an eigenspace with cardinality *k > *1 to be the projection of that object on the eigenspace:

e^(i)=∑j=1kej(i)2
 MathType@MTEF@5@5@+=feaafiart1ev1aaatCvAUfKttLearuWrP9MDH5MBPbIqV92AaeXatLxBI9gBaebbnrfifHhDYfgasaacH8akY=wiFfYdH8Gipec8Eeeu0xXdbba9frFj0=OqFfea0dXdd9vqai=hGuQ8kuc9pgc9s8qqaq=dirpe0xb9q8qiLsFr0=vr0=vr0dc8meaabaqaciaacaGaaeqabaqabeGadaaakeaaieqacuWFLbqzgaqcaiabcIcaOiabdMgaPjabcMcaPiabg2da9maakaaabaWaaabCaeaacqWFLbqzdaWgaaWcbaGaemOAaOgabeaakiabcIcaOiabdMgaPjabcMcaPmaaCaaaleqabaGaeGOmaidaaaqaaiabdQgaQjabg2da9iabigdaXaqaaiabdUgaRbqdcqGHris5aaWcbeaaaaa@403A@

where {**e**_1_, **e**_2_, ..., **e**_*k*_} is an orthonormal basis for the eigenspace. I.e. this is the total prominence value assigned by that eigenspace. Note **ê**(*i*) is equal to the maximal projection over all vectors within the eigenspace. We therefore effectively assign each node's prominence to be equal to the maximum prominence assigned to that node in *any *vector in the eigenspace.

It should also be noted that all existing methods for ranking nodes in large data graphs (such as the web) focus on just the principal eigenspace when assigning prominence values. However, the connection between prominence assignments and the spectra of appropriate matrices suggests that non-principal eigenvectors may also convey useful notions of prominence. For example, Kleinberg [14] notices that different eigenvectors emphasize webpages with different interpretations of the query term. We explore methods for combining the information from multiple eigenspaces and evaluate their usefulness in ranking documents in the 'Discussion' section.

### Search strategies: global graph vs. focused subgraphs

We investigate the performance of the prominence models described above. Our ultimate goal is to provide a sensible ranking of results from queries to the Biozon database. We consider two general strategies for generating and then ordering these result sets: The **focused subgraph method **(referred to also as the 'local method') first creates a subgraph of Biozon consisting of nodes that satisfy the query and their immediate neighbors (the exact procedure is described next). Prominence values are then assigned to the nodes of the subgraph using one of the above models, and nodes are ranked by descending prominence. The **global method, **on the other hand, first uses one of the models to assign prominence values to every node in the Biozon graph. Then, for a given search, nodes satisfying the query are extracted and then ordered by descending prominence.

#### Generating instance sets – the query subgraph

The local method starts by generating a specific set of relevant instances, and then ranks the instances by exploring the spectral properties of the corresponding connectivity matrix. Given a query term *Q *(such as ubiquitin) over a data type *T *(e.g. protein sequences) we run the following procedure to generate the **query instance set**

1. Initialize the instance set **S_1 _**to be all instances of type *T *containing the text *Q *in their definition field (in this study we focused on the definition field, but the search can be extended to other fields).

2. Add to **S_1 _**any object (of any data type *T'*) that is directly related to an object in **S_1 _**(note that in the case of proteins, the similarity relation can bring in other proteins directly related to those already in **S_1_**).

3. Initialize **S_2 _**to include any object of data type *T' ≠ T *that contains the text *Q *in its definition field.

4. Add to **S_2 _**all objects of type *T *which are not in **S_1 _**but are directly related to objects (of any data type) in the set **S_2_**.

5. Merge **S_1 _**and **S_2 _**to form the complete instance set **S**.

This procedure resembles the procedure introduced in [14]. However it has been modified to account for heterogeneous data types. Specifically, step 4 serves to extend the instance set to other possibly relevant instances of the query data type. On the other hand, extensions with regard to other data types are more restricted to avoid shift of biological context (steps 2,3). In some cases we introduce additional constraints. For example, when querying for proteins we only consider sequences that are at least 20 amino acids long. This is to exclude very short peptides that are linked to many DNA sequences but have limited biological significance.

The resulting subgraph of the Biozon data graph is called the **query subgraph. **The nodes of the query subgraph are the objects in the query instance set. The edges of the query subgraph are obtained by projecting the complete Biozon data graph onto the instance set **S**. An example of a focused subgraph is given in Fig. 1. The composition of the focused subgraph for selected queries is given in Table 5.

#### The extended global method

The naive implementation of the global method appears to be too restrictive when compared to the local method. The local method is not limited to entities defined by the query term; it may return an entity *related *to another entity which is defined by the query term. This allows the local method to propagate information to uncharacterized objects and potentially increase the number of valuable results. However, the global method has a better response times since it relies on precomputed prominence values. To compensate for the loss of information inherent in the global method without reducing responsiveness, we introduce a third strategy for generating results. The **extended global **method is a variation of the global method which utilizes information in neighboring entities to bring forward uncharacterized entities as follows:

As with the global method, a prominence model is applied to the Biozon data graph once and for all to assign prominence values to all documents. Given a query term *Q *over data type *T*

1. Search for all documents that match the query term *Q*. Denote that set by **S_0_**.

2. Collect the top-scoring documents of **S_0 _**until encountering at least *N *objects of data type *T *or until exhausting **S_0 _**(in our experiments, *N *is set to 50). The resulting set, **S_1_**, contains documents of all types.

3. Separate **S_1 _**into **S_2 _**(documents of type *T*) and **S_3 _**(documents of type ≠ *T*).

4. Initialize **S **to **S_2_**.

5. Add to **S **all objects of type *T *related to any object in **S_3_**.

6. The set **S **contains only objects of type *T*, some of which match the query term. Reorder the elements of **S **based on their global prominence value.

### The distributions of eigenvalues and prominence values

**Figure 9:**
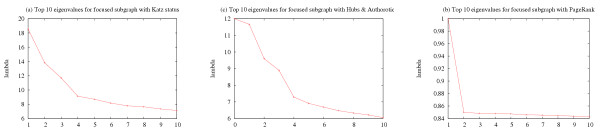
**Top 10 eigenvalues of different connectivity matrices**. (a) Hybrid Katz's Status (b) Hubs & Authorities (c) PageRank. The eigenvalues were computed for matrices that were generated from focused subgraphs with the protein query ubiquitin. Similar results were observed for other queries.

We study the distributions of the eigenvalues of the different prominence models' connectivity matrices. This analysis can help determine how many different eigenvectors should be used when ranking instance sets (see 'Discussion'). In general, we observe that the eigenvalues produced by sparse models all display similar decay patterns across different queries (Fig. 9a and Fig. 9b). PageRank, on the other hand, usually produces one relatively large eigenvalue while the rest of the eigenvalues all have very similar smaller values (Fig. 9c).

**Figure 10:**
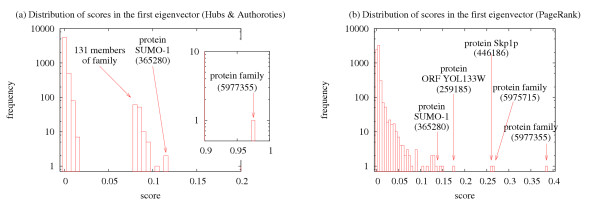
**Distribution of prominence values in eigenvectors**. (a) Hubs & Authorities (b) PageRank. The prominence values were derived from the principal eigenvector of the connectivity matrix that was generated from focused subgraph with the protein query ubiquitin. Sparse models assign high value to the protein family ubiquitin-protein ligase, ubiquitin-activating enzyme (docid: 5977355), and medium values to its 131 members. All other nodes are given values less than 0.02. Similar patterns of prominence values are observed in other eigenspaces and for other queries. On the other hand, PageRank consolidates the top-scoring documents from multiple eigenspaces of the other methods, resulting in a smoother distribution of prominence values.

We examined the prominence vectors produced by the different prominence models on focused subgraphs, and studied the distribution of values within these vectors. We observe that for a given prominence model, the properties of the distribution of prominence values within an eigenvector are fairly consistent both across different query terms and across the different eigenvectors of a particular matrix. However, these distributions do differ between different models. We found that the distributions of prominence values of sparse models are all nearly identical. Often, there is one document that gets a very high prominence value, while the vast majority of nodes receive very small values. Between these two extremes we generally observe a significant number of nodes which are often clustered around intermediate values but are sometimes evenly distributed in a small range (see Fig. 10a). With the PageRank method, the distributions of the prominence values display similar characteristics. Most notably, the vast majority of documents receive relatively small prominence values. However, PageRank seems to collect into its principal eigenspace all the top scoring documents which the other models report in separate eigenspaces. Consequently, the distribution of higher valued documents is much smoother, and the number of documents at a given value appears to be inversely proportional to that value (Fig. 10b). These observations mesh with our previous observations in section 'Examples'.

We also studied the distribution of prominence values in the *principal *eigenvectors produced by the various prominence models when considering the *global *graph. As one would expect, since only the relative size of the graph has changed, the general shape of these distributions is basically the same as those for the smaller focused subgraphs. However, for Hubs & Authorities there is not a single high-valued node in the principal component. There is, though, a cluster of about 9000 items assigned values of about 0.004. These mostly correspond to the members of the largest protein family in Biozon (Cytochrome C oxidase), which has about that many members.

It should be noted that only a small fraction of prominence values are actually relevant in each eigenspace. However, *a priori *it is unclear which prominence values should be considered significant, since every eigenvector spans the whole query graph and all documents are assigned prominence values in each eigenspace. The majority of the documents are assigned very small prominence values (close to zero), but in some eigenspaces values of 0.05 can be considered significant while in others they are not.

**Figure 11:**
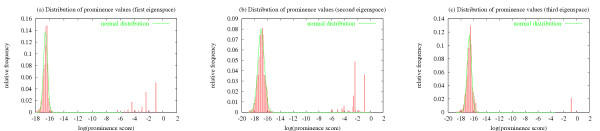
**Significance of prominence values**. Prominence values were assigned by the Eigenvector Centrality model applied to the focused subgraph with the protein query cancer. Distributions are shown for the first, second and third eigenspaces. The distributions of low-scoring nodes are modeled by normal distributions. Of the 1977 nodes in the focused subgraph, exactly 264 are more than 5 standard deviations apart from the mean of the normal distribution, in the first and second eigenspaces, and 44 in the third eigenspace. Note that these numbers correspond to the size of the connected components that are mapped to these eigenspaces (see section 'Eigenspaces and connected components'). The top scoring nodes in both the first and second eigenspaces are drawn from the largest CC, but different groups of documents are assigned the most significant values in each eigenspace.

To address this problem we model the distribution of low-scoring documents (see Fig. 11) and use that distribution to estimate the significance of outliers. For all practical purposes this distribution can be approximated by a normal distribution. To estimate the parameters of the normal distribution automatically we apply a simple iterative procedure. In the first iteration all data points are used to estimate parameters. The initial estimate is used to assign probabilities to all documents and documents that are more than 3 standard deviations apart from the mean are eliminated. The procedure is repeated until convergence (no documents are eliminated) or until a maximal number of 10 iterations is reached.

### Variations on prominence models

This section focuses on prominence models that are tailored specifically to the biological knowledge domain. We propose and test several variations that use the structure of the Biozon graph and the different data and relation types to improve the ranking.

### Forced directed graphs

In the Biozon data graph certain relations have a clear causal interpretation. For example, a DNA sequence encodes for a protein sequence and a protein sequence manifests itself as a structure. In general, although all relations in Biozon are undirected, each relation can be associated with a default direction (defined by the active voice name of the relation). However, adhering to the default directionality is arbitrary and disregards the equally informative reverse relations. Therefore, the undirected graph was chosen early on as the default setup in this study. Undirected graphs are also preferred because they have symmetric adjacency matrices and thus real eigenvalues. Nevertheless, there are certain settings in which directed graphs are useful.

If the default directions of edges in Biozon were used to construct a directed adjacency matrix for the Hubs & Authorities model, then certain types would be naturally viewed as hubs and other types as authorities, regardless of the target query type. For example, DNA sequences would be candidate hubs with respect to protein sequences, and protein sequences would be candidate hubs for structures. This information can be useful when sorting out the contributions of different entities to the ranking of others and when tracking prominence propagation. However, with the default directionality, certain object types can never contribute to authority scores of other object types. For example, structures will not contribute any information to the authority scores of protein sequences since the default direction of edges is from sequences to structures. Yet, each relation can be reversed, and this is basis for the **forced directed graph. **In this setting, all edges are directed toward a particular data type.

Suppose we are interested in assigning prominence to nodes of a specific data type *T *in a focused subgraph. To confer as much authority on that data type as possible, we re-organize the graph. Every edge (*u, v*) such that *u *is of data type *T *and *v *is of data type *T' ≠ T *is replaced with its reversed edge (*v, u*). Thus, in the resulting graph an edge involving a node of type *T *will point towards the document of type *T*. All edges (*u, v*) such that both *u *and *v *are not of data type *T *are replaced with two directed edges. In this setup, when querying for proteins, structures will contribute to protein authority scores. [In the global setting we do not know ahead of time what data type the user will query. Therefore, we build a forcing adjacency matrix for each data type and assign prominence according to a node's authority score in its corresponding data type's forcing adjacency matrix. Other ideas for combining forcing adjacency matrices for different data types still need to be investigated.]

Our experiments with forced focused subgraphs did not indicate a significant increase in overall performance compared to undirected graphs. However, the forced focused subgraph method is advantageous because it produces a clear and sensible distinction between hubs and authorities, where authorities are elements of the query data type and hubs are elements of other data types that contribute to authority scores. For example, in Table 6 we list the top hubs and authorities for the query ubiquitin in forced subgraph mode. The top hubs for the ubiquitin and ubiquitin-like proteins are either protein families or interactions with other ubiquitin related proteins that are interconnected to many other entities. Interestingly, the ranking brings forward a ubiquitin protein that interacts with the tumor suppressor protein p53 which is connected to many other entities.

**Table 6:** Top hubs & authorities objects in the principal eigenspace of a forced subgraph.

	Score	Definition	Docid
**Authority:**	0.119 (A)	Ubiquitin-like protein SUMO-1	365280
**Hubs:**	0.98 (H)	Protein family, ubiquitin-protein ligase	5977355
	0.01 (H)	Interaction with p53	60851937
	0.01 (H)	Interaction with ubiquitin-like protein SMT3C	60845291
**Authority:**	0.117 (A)	Ubiquitin-protein ligase RSP5	1026628
**Hubs:**	0.98 (H)	Protein family, ubiquitin-protein ligase	5977355
	0.01 (H)	Interaction with ubiquitin-conjugating enzyme MMS2	60849549
	0.01 (H)	Interaction with ubiquitination pathway protein BUL1	60889006

Results are reported for the Hubs & Authorities model using the query ubiquitin with 'proteins' as the target data type. The top two ranked documents are shown, along with their authority scores. Below each is a list of the three neighbors with the highest hub scores (those neighbors that contribute most to the authority score of the original object). All interactions were contributed by DIP [6] but are available to the public only from the DIP website.

### The effect of similarity data

Similarity information plays a fundamental role in the analysis of biological entities and especially macromolecules. For example, analysis of a new gene almost always starts with a database search followed by a careful examination of close homologs. Depending on the degree and the extent of the similarity, properties of the new gene can often be inferred from its homologs. Similarly, experimentally determined protein-protein interactions in one organism can be extrapolated to other organisms if homologous proteins can be found.

Biozon integrates similarity relationships into its internal schema, thus allowing us to exploit an enormous amount of knowledge in an unprecedented way. The total number of significant similarities in Biozon, based on both protein sequences and structures, exceeds 2.5 billion. In most cases these similarity relations indicate strong homology and similar functions.

So far our study has ignored these relations because they increase the complexity of the analysis by orders of magnitude. However, this information can be extremely useful in characterizing new objects since many functional descriptors can propagate between similar entities. We tested the effect of similarity data on focused subgraphs for a subset of our queries. We repeated the process of generating focused subgraphs, this time considering also similarity relations between entities with *evalue *≤ 10^-10^. (Increasing the threshold, i.e. including less significant similarities, resulted in many more similarity relations to the point that we were not able to build the subgraphs.) We then applied the Hubs & Authorities prominence model, and the results were evaluated as before using the *UROC *measure. Our results indicate that similarity data can be useful for prominence calculations; however, the results are not consistent. Two opposing examples are given in Table 7. For one query (stromelysin) similarity data clearly improves the ranking, while for the other (autoimmune) the opposite effect is observed. This behavior is most likely the result of two factors: redundancy and localization (or lack thereof). Protein sequence databases are highly redundant, and some proteins are related to hundreds or thousands of other very similar proteins. While overall useful, these relations can overwhelmingly dominate other types of relations when analyzing prominence. Moreover, the definitions of proteins similar to a particular protein can be quite diverse, introducing a high level of noise. For nodes with extremely high in-degrees (due to many similar proteins), this often results in less coherent subgraphs. The second problem is even more substantial. Similarity data is localized (i.e. two sequences usually share a similar *subsequence*). In other words, similarity data should be used carefully when extrapolating the properties of one entity to another, and only the functional descriptors that are associated with these subsequences can be reliably propagated between the sequences. However, functional descriptors that are available in sequence databases are almost never localized, and therefore it is difficult to discern which descriptors should be propagated and which should not. When the coherence among related entities is weak one might suspect that localization is to blame.

**Table 7:** The effect of similarity data.

Query Term	Query Type	Prominence Model	Focused Subgraph	Average No. Neighbors	Average Consistent Neighbors	Average Ratio	*Q*(**R**)	*UROC *(**R**)
autoimmune	protein	Hubs & Authorities	With Sim	1413.76	1.98	0	439	11773
			No Sim	69.8	4.64	0.13	2062	57865
stromelysin	protein	Hubs & Authorities	With Sim	338.52	27.98	0.083	10348	263293
			No Sim	214.91	19.3	0.22	6690	181282

These quality results were calculated for the stromelysin and autoimmune focused subgraphs when searching for proteins. The Hubs & Authorities values were computed using the Max scoring method. To compare these results to our previous results we recomputed all performance measures for the focused subgraphs that include similarity relations, but using the ranking that was produced without considering these relations. Interestingly, when using similarity data, the top scoring entity for the 'stromelysin' query is a protein (docid 986092) that does not contain the query term in its definition, nor do the DNA sequences, the UniGene clusters, and the enzyme family that are related to this protein. However, this protein, membrane type 5 matrix metalloproteinase, is significantly similar to many stromelysin proteins.

### The biological significance of functional links

Not all links between entities in Biozon carry the same functional weight. Moreover, biological databases can be heavily biased or redundant. As a result, the functional links in Biozon are not equally meaningful or biologically significant. For example, a protein may be linked to a long DNA sequence that encodes for many genes. The relevant information that the DNA sequence carries with respect to that particular protein is limited, and the longer the DNA (and the more genes it encodes), the less specific is the information. On the other hand, the same protein might be linked to an interaction that is essential for a specific biological process. This relation clearly carries more weight than that with the DNA sequence when characterizing the biological context of the protein. These factors should be taken into account when analyzing the link structure of the Biozon data graph. A possible solution is to take a knowledge-based approach and weight the relations based on their biological significance.

To tailor the prominence models to the specific domain of biological data, we test a variation where different types of edges are weighted differently. Instead of a 1/0 adjacency matrix we associate weights that we believe reflect the importance of association, and generate appropriate connectivity matrices based on the weighted adjacency matrix. For example, edges incident to a particular DNA sequence that encodes for multiple proteins are weighted such that the weight of each edge (*DNA, Protein*) is 1/*n *where *n *is the total number of proteins encoded by that DNA sequence. However, the reverse edges are still assigned a weight of 1, thus breaking the symmetry that was inherent to the original Biozon data graph. Similarly, if multiple protein structures are mapped to the same protein sequence (this is quite often the case, as the protein structure can be studied under different experimental conditions), then each edge (*Structure, Protein*) is assigned a weight 1/*n *where *n *is the total number of structures associated with the protein sequence. The reverse edges, on the other hand, are assigned a weight of 1. The connectivity of a node does not always decrease the weights of its outgoing edges. For example, a protein family object can point to many proteins, and each edge is assigned a weight of 1. This is to indicate that the information summarized in the definition of a protein family is highly reliable compared to the definitions of the individual proteins. On the other hand proteins that are associated with a protein family are usually very similar and the information contained in their records can be highly redundant. Therefore, with our weighting schema the sum of the weights associated with family members is one, and their collective contribution to the family's prominence value is equal to the average prominence value among them. Similarly, we weight the contributions of all similarity relations incident to a protein sequence such that their collective weight is 1. (If a protein is linked to *n *other proteins through similarity relations then each one is weighted 1/*n*. Alternatively, the weight can be divided based on the *pvalue *of the similarity that is derived from the *evalue *of the similarity score by the transformation *pvalue *= 1 - exp(-*evalue*) as in [26].)

This simple schema can be extended to include also descriptor documents that we have ignored so far. With the same reasoning, descriptors should have different weights than objects pointing to another object because descriptors are redundant. On the other hand they reinforce each other. A possible solution is to set the weights of descriptors such that they sum to 1.

As with similarity data, here too we observe inconsistent trends (results not shown). While in some cases edge weighting clearly improves the results, in others it worsens their quality, and further study is necessary to converge to a stable and consistent weighting scheme. Nevertheless, these results suggest that the biological knowledge domain requires more fine tuning than the WWW, based on the different functional meanings of the nodes and the relations between them.

## Authors' contributions

GY conceived of the study, devised the evaluation methodology and designed the experiments. PS studied and implemented the prominence models and developed the variation on Katz's status and the forced directed graphs. TI studied the prominence models and implemented and tested the evaluation scripts and variations thereof. PS and TI ran the experiments and analyzed the results. All authors contributed to the paper.
